# Genomic and Developmental Models to Predict Cognitive and Adaptive Outcomes in Autistic Children

**DOI:** 10.1001/jamapediatrics.2025.0205

**Published:** 2025-04-21

**Authors:** Vincent-Raphaël Bourque, Zoe Schmilovich, Guillaume Huguet, Jade England, Adeniran Okewole, Cécile Poulain, Thomas Renne, Martineau Jean-Louis, Zohra Saci, Xinhe Zhang, Thomas Rolland, Aurélie Labbé, Jacob Vorstman, Guy A. Rouleau, Simon Baron-Cohen, Laurent Mottron, Richard A. I. Bethlehem, Varun Warrier, Sébastien Jacquemont

**Affiliations:** 1 CHU Sainte-Justine Pediatric Hospital and Research Centre, Université de Montréal, Montréal, Québec, Canada; 2 Autism Research Centre, Department of Psychiatry, University of Cambridge, Cambridge, United Kingdom; 3 Faculty of Medicine and Health Sciences, McGill University, Montréal, Québec, Canada; 4 Département de Psychiatrie, Université de Montréal, Montréal, Québec, Canada; 5 Département de Pédiatrie, Université de Montréal, Montréal, Québec, Canada; 6 Département de Biochimie et Médecine Moléculaire, Université de Montréal, Montréal, Québec, Canada; 7 Human Genetics and Cognitive Functions, Institut Pasteur, UMR3571 CNRS, Paris, France; 8 HEC Montréal, Montréal, Québec, Canada; 9 The Hospital for Sick Children, University of Toronto, Toronto, Ontario, Canada; 10 The Neuro, Montreal Neurological Institute-Hospital, McGill University, Montréal, Québec, Canada; 11 Centre de Recherche, Évaluation et Intervention en Autisme (CRÉIA), Rivière-des-Prairies Hospital, CIUSSS du Nord-de-l’île-de-Montréal, Montréal, Québec, Canada; 12 Department of Psychology, University of Cambridge, Cambridge, United Kingdom

## Abstract

**Importance:**

Although early signs of autism are often observed between 18 and 36 months of age, there is considerable uncertainty regarding future development. Clinicians lack predictive tools to identify those who will later be diagnosed with co-occurring intellectual disability (ID).

**Objective:**

To predict ID in children diagnosed with autism.

**Design, Setting, and Participants:**

This prognostic study involved the development and validation of models integrating genetic variants and developmental milestones to predict ID. Models were trained, cross-validated, and tested for generalizability across 3 autism cohorts: Simons Foundation Powering Autism Research (SPARK), Simons Simplex Collection, and MSSNG. Autistic participants were assessed older than 6 years of age for ID. Study data were analyzed from January 2023 to July 2024.

**Exposures:**

Ages at attaining early developmental milestones, occurrence of language regression, polygenic scores for cognitive ability and autism, rare copy number variants, de novo loss-of-function and missense variants impacting constrained genes.

**Main Outcomes and Measures:**

The out-of-sample performance of predictive models was assessed using the area under the receiver operating characteristic curve (AUROC), positive predictive values (PPVs), and negative predictive values (NPVs).

**Results:**

A total of 5633 autistic participants (4574 male [81.2%]) were included in this analysis. On average, participants were diagnosed with autism at 4 (IQR, 3-7) years of age and assessed for ID at 11 (8-14) years of age, with 1159 participants (20.6%) being diagnosed with ID. The model integrating all predictors yielded an AUROC of 0.653 (95% CI, 0.625-0.681), and this predictive performance was cross-validated and generalized across cohorts. This modest performance reflected that only a subset of individuals carried large-effect variants, high polygenic scores, or presented delayed milestones. However, combinations of genetic variants that are typically not considered clinically relevant by diagnostic laboratories achieved PPVs of 55% and correctly identified 10% of individuals developing ID. The addition of polygenic scores to developmental milestones specifically improved NPVs rather than PPVs. Notably, the ability to stratify ID probabilities using genetic variants was up to 2-fold higher in individuals with delayed milestones compared with those with typical development.

**Conclusions and Relevance:**

Results of this prognostic study suggest that the growing number of neurodevelopmental condition–associated variants cannot, in most cases, be used alone for predicting ID. However, models combining different classes of variants with developmental milestones provide clinically relevant individual-level predictions that could be useful for targeting early interventions.

## Key Points

**Question:**

What is the predictive performance of combining genetic variants and developmental milestones to predict intellectual disability (ID) in autistic children?

**Findings:**

In this prognostic study including 5633 autistic participants, a model combining 5 classes of genetic variants and early developmental milestones yielded an area under the receiver operating characteristic curve of 0.65, with this predictive performance cross-validated and generalized across cohorts. The model yielded positive predictive values of 55%, accurately identifying 10% of ID cases; the ability to stratify ID probabilities using genetic variants was up to 2-fold higher in individuals with delayed milestones compared with those with typical development.

**Meaning:**

Results suggest that models that integrate genetic and developmental information could be implemented in clinical settings to help anticipate developmental trajectories in autism and target early interventions.

## Teaser

This prognostic study evaluates the predictive performance of combining genetic variants and developmental milestones to predict intellectual disability in autistic children.

## Introduction

Parents of children diagnosed with autism in early childhood invariably question their child’s future cognitive and adaptive development: will they be able to communicate, interact socially, and live independently?^1^

Early signs of autism, including differences in social communication and repetitive or restricted behavior, often manifest around 18 months of age.^2,3^ These first concerns may be accompanied by delays in language development, and in a more limited proportion of individuals, by delays in motor milestones.^4^ Diagnoses of autism made at 18 to 36 months are stable in most cases,^5,6,7^ yet significant uncertainty remains regarding future development.^8^ Autistic individuals can later display a broad diversity of strengths^9^ and disabilities, which might not directly correspond to their early developmental presentation.^10^ This is exemplified by intellectual disability (ID), a clinical diagnosis characterized by impairments in both cognitive ability and adaptive functioning, which occurs in approximately 10% to 40% of autistic individuals^11,12^ and can only be diagnosed with relative certainty after age 6 years.^13,14,15,16^

Interventions have been reported to benefit some autistic individuals,^8,17^ guided by general principles of maximizing potential, minimizing barriers, and optimizing the person-environment fit.^18^ However, the current wait-and-see approach^19^ often overlooks a child’s specific strengths and challenges until there is a significant mismatch^20,21^ between the individual’s abilities, the environmental demands, and the provided support,^18^ which may result in higher levels of stress, school failure, and social misunderstandings. As such, anticipating cognitive and adaptive profiles may be key to determining what specific interventions are best offered, to whom, when, and at what intensity.^22^

Recent genetic research suggests a potential for polygenic scores (PGS) and rare genetic variants—including copy number variants (CNVs; deletions or duplications) and single nucleotide gene-disrupting variants—as predictors of cognitive and adaptive profiles in autistic individuals.^23,24,25^ However, even strong statistical associations derived from group-level comparisons do not necessarily translate into clinically relevant individual-level predictions.^26^ Currently, clinicians attempt to intuitively integrate genetic findings with the surveillance of developmental milestones^8^ but thus far without the assistance from any predictive models, as are increasingly used in other medical fields.^27,28,29,30,31^

Here, we aimed to develop and validate models integrating genetic variants and early developmental milestones to predict the probability of developing ID in toddlers and young children receiving a diagnosis of autism.

We asked the following: (1) Does combining different classes of genetic variants improve ID prediction? (2) Does the integration of genetic variants with developmental milestones outperform milestones alone? (3) What are the cognitive and adaptive dimensions more precisely predicted by genetic variants and developmental milestones?

## Methods

### Ethics and Reporting Standards

This study was approved by the CHU Sainte-Justine Research Centre institutional review board. All participants provided written informed consent. This study adhered to the Transparent Reporting of a Multivariable Prediction Model for Individual Prognosis or Diagnosis (TRIPOD) reporting guidelines^32^ and the Polygenic Risk Score Reporting Standards (PRS-RS).^33^

### Sample Selection

We included participants from 3 cohorts: the Simons Foundation Powering Autism Research (SPARK)^34^ version WES1-2-3, Simons Simplex Collection (SSC),^35^ and MSSNG.^36^ Inclusion criteria were as follows: a professional diagnosis of autism spectrum disorder according to *Diagnostic and Statistical Manual of Mental Disorders* Fifth Edition, Text Revision, or corresponding categories from previous editions, based on a caregiver report or self-report; genetic data from the proband and both parents meeting quality control; genetically inferred European ancestry; documented information on ID; and age at least 6 years for greater stability of this assessment.^37^ Analyses of developmental milestones were done in the subset of individuals for whom all milestones were available.

### Outcome

In the SPARK cohort, ID was determined based on a caregiver report of a professional diagnosis (eMethods in Supplement 1). We validated that cognitive and adaptive measures, where available from medical records, were strong predictors of this report (eTable 1 in Supplement 2). In the SSC and MSSNG cohorts, ID was inferred from nonverbal IQ data using a threshold below 70.^38^ In a subsample of the SSC cohort where data were consistently available, we conducted a secondary analysis on the subscales from the IQ and the Vineland Adaptive Behavior Scales.^39^

### Genetic and Developmental Predictors

The complete procedure for calling and processing genetic variants is detailed in the eMethods in Supplement 1. Briefly, using feature selection algorithms, we retained PGS for cognitive ability^40^ and autism^41^ as the combination most predictive of ID in the training cohort, among 12 PGS (eTable 2 in Supplement 2), previously selected^41^ from 234 genome-wide association study summary statistics tested for their genetic correlation with autism.^41^

Across 4 classes of rare variants (deletions, duplications, de novo loss of function [LOF] and de novo missense variants), we used a gene-based scoring strategy, assigning scores to each individual based on the count of rare variants, within each class, affecting constrained genes. Constrained genes are relatively intolerant to variation, and therefore, pathogenic variants in these genes are more likely to have deleterious effects on development; these were defined with LOF observed/expected upper bound fraction (LOEUF) less than 0.35,^42,43,44^ which represents 2971 genes, genome-wide. In addition, we evaluated a smaller set of 285 genes,^45^ potentially more specifically associated with developmental disorders (DDs). Developmental milestones (motor, language and toileting) were assessed based on retrospective reports by caregivers.

### Framework for Model Training and Evaluation

To determine the individual and cumulative predictive contributions of genetic and developmental variables, we used multiple logistic regression, sequentially adding variables in a predetermined order (eMethods in Supplement 1). Using 10-fold cross-validation in the SPARK cohort, we systematically trained the models and assessed their predictive performance on unseen data. By averaging the performance metrics across all folds, this method^46^ provides robust estimates, more reflective of the out-of-sample predictive performance.^47^ Finally, we tested out-of-sample prediction of the models trained on the complete SPARK sample to generalize on the SSC and MSSNG samples. Metrics for assessing the models’ performance are described in eAppendix in Supplement 1: the areas under the receiver operating characteristic (AUROC) curve,^48^ as well as positive predictive value (PPV)–sensitivity^28^ and negative predictive value (NPV)–specificity curves.

### Statistical Analysis

CIs on performance metrics were computed using bootstrap methods with 10 000 iterations. In addition, the statistical significance of improvement in performance when adding new predictors to the model was assessed using the likelihood-ratio test.^49^ *P* values were adjusted for multiple comparisons using the Benjamini-Yekutieli method (statistical significance 2-sided *P* < .05). Analyses were conducted with RStudio, version 4.3.2 (Posit PBC), and Scikit-Learn^50^ with Python, version 3.11.6 (Python Software Foundation). Study data were analyzed from January 2023 to July 2024.

## Results

### Descriptive Statistics of the Cohorts

**Figure 1.**
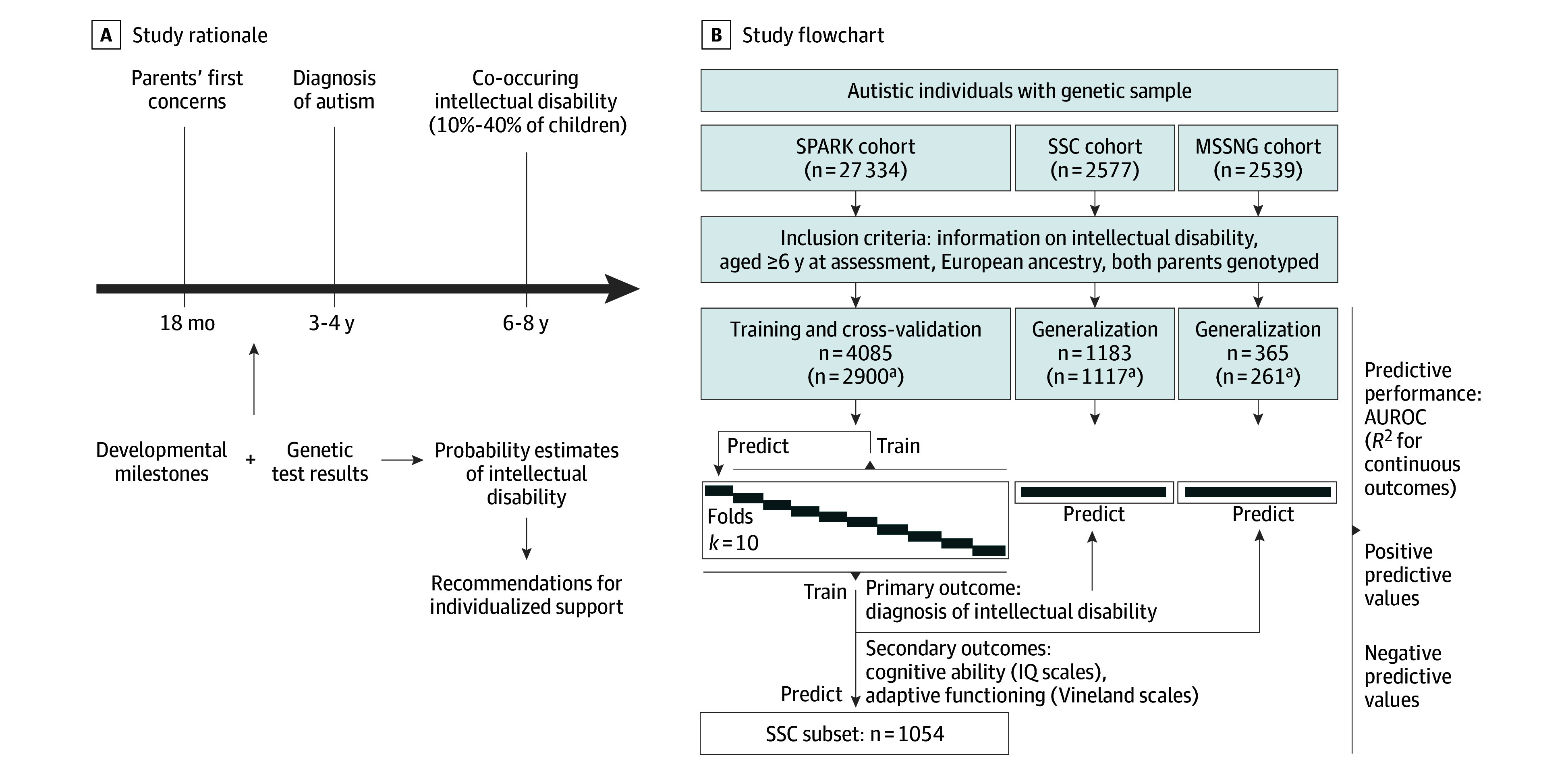
Schematic Overview of the Study A, Study rationale. There is often a long period of uncertainty between parents’ initial concerns, an autism diagnosis, and the identification of co-occurring intellectual disability (ID) in 10% to 40% of autistic children. Genetic testing is frequently conducted at or before the diagnosis of autism. Therefore, predictive models that integrate genetic testing results with developmental milestones could help clinicians provide parents with accurate information about their child’s expected developmental trajectory such as to offer the child appropriate support. B, Study flowchart. We trained models to predict ID in the Simons Foundation Powering Autism Research (SPARK) cohort, estimating their out-of-sample predictive performance using cross-validation. Additionally, we evaluated the generalizability of these models in the Simons Simplex Collection (SSC) and MSSNG cohorts. AUROC indicates area under the receiver operating characteristic curve. ^a^Individuals with complete milestones data.

A total of 5633 autistic individuals (median [IQR] age, 11 [8-14] years; 1059 female [18.8%]; 4574 male [81.2%]) were included across the 3 cohorts (Figure 1), and their characteristics are detailed in the Table.^42,45,51,52^ Participants were diagnosed with autism at a median age of 4 years, even though their parents reported concerns starting from 18 months of age. ID occurred in 13.7% to 22.0% of individuals, depending on the sample, and was assessed or reported at a median age of 10 to 11 years.

**Table.** Participant Characteristics^a^

Characteristic	Cohort used for training and cross-validation, SPARK (n = 4085)	Cohorts used for generalization	
		SSC (n = 1183)	MSSNG (n = 365)
Profile of participants			
Sex, No. (%)			
Female	825 (20.2)	152 (12.8)	82 (22.5)
Male	3260 (79.8)	1031 (87.2)	283 (77.5)
Age at parents’ first concern, median (IQR), mo	18.0 (12.0-30.0)	18.0 (14.0-30.0)	18.0 (12.0-30.0)
Age at diagnosis of autism, median (IQR), y	4.25 (2.83-7.00)	NA	NA
Age at ID assessment, median (IQR), y	11.3 (8.42-14.9)	9.67 (7.92-12.8)	10.1 (8.21-12.9)
Outcome			
Diagnosis of ID, No. (%)	849 (20.8)	260 (22.0)	50 (13.7)
Developmental predictors, median (IQR)			
Age at independent walking (general population 90th percentile: 18 mo)	13.0 (12.0-15.0)	12.0 (11.0-15.0)	13.0 (12.0-16.0)
Delayed, No. (%)	515 (16.7)	122 (10.4)	47 (16.8)
Age at first word (general population 90th percentile: 15 mo)	14.0 (11.0-24.0)	18.0 (12.0-30.0)	18.0 (12.0-36.0)
Delayed, No. (%)	1429 (48.6)	737 (65.4)	178 (66.9)
Age at first phrase (general population 90th percentile: 24 mo)	24.0 (18.0-42.0)	36.0 (24.0-48.0)	36.0 (24.0-48.0)
Delayed, No. (%)	1703 (60.3)	843 (78.5)	192 (77.4)
Language regression (occurs on average at 21.8 mo in autism), No. (%)^51^	710 (22.7)	181 (15.4)	47 (16.6)
Bladder control, mo	42.0 (36.0-54.0)	43.0 (36.0-54.0)	45.5 (36.0-54.0)
Bowel control, mo	48.0 (36.0-60.0)	48.0 (37.0-60.0)	48.0 (36.0-60.0)
Carriers of genetic variants, No. (%)			
Deletions			
Impacting constrained genes	135 (3.3)	27 (2.3)	4 (1.1)
Impacting DD genes	32 (0.8)	5 (0.4)	2 (0.5)
Duplications			
Impacting constrained genes	195 (4.8)	52 (4.4)	17 (4.7)
Impacting DD genes	27 (0.7)	7 (0.6)	4 (1.1)
De novo missense variants with MPC ≥2			
Impacting constrained genes	127 (3.0)	48 (4.1)	12 (3.3)
Impacting DD genes	63 (1.5)	25 (2.1)	6 (1.6)
De novo LOF variants			
Impacting constrained genes	291 (7.1)	135 (11.4)	27 (7.4)
Impacting DD genes	137 (3.4)	46 (3.9)	9 (2.5)
Any of the aforementioned variants			
Impacting constrained genes	707 (17.3)	244 (20.6)	59 (16.2)
Impacting DD genes	254 (6.2)	82 (6.9)	21 (5.8)

Abbreviations: DD, developmental disorder; ID, intellectual disability; LOF, loss of function; MPC, missense polyphen constraint; NA, not assessed; SPARK, Simons Foundation Powering Autism Research; SSC, Simons Simplex Collection.

^a^ Autistic individuals were included from 3 distinct cohorts: SPARK, SSC, and MSSNG. For each milestone, we present the median (IQR) and the percentage delayed as compared with the 90th general population percentile. Constrained genes (2971 genes genome wide) are intolerant to gene variants and, therefore, pathogenic variants in these genes likely to have deleterious effects on development.^42^ DD genes represent a smaller set of genes (285 genes genome wide) previously associated with severe developmental disorders.^45^ Delayed milestones were defined based on the 90th general population percentile.^52^

### Predictive Value of Combining Different Classes of Genetic Variants

#### Evaluating Genetic Variants With AUROC Curve Analysis

**Figure 2.**
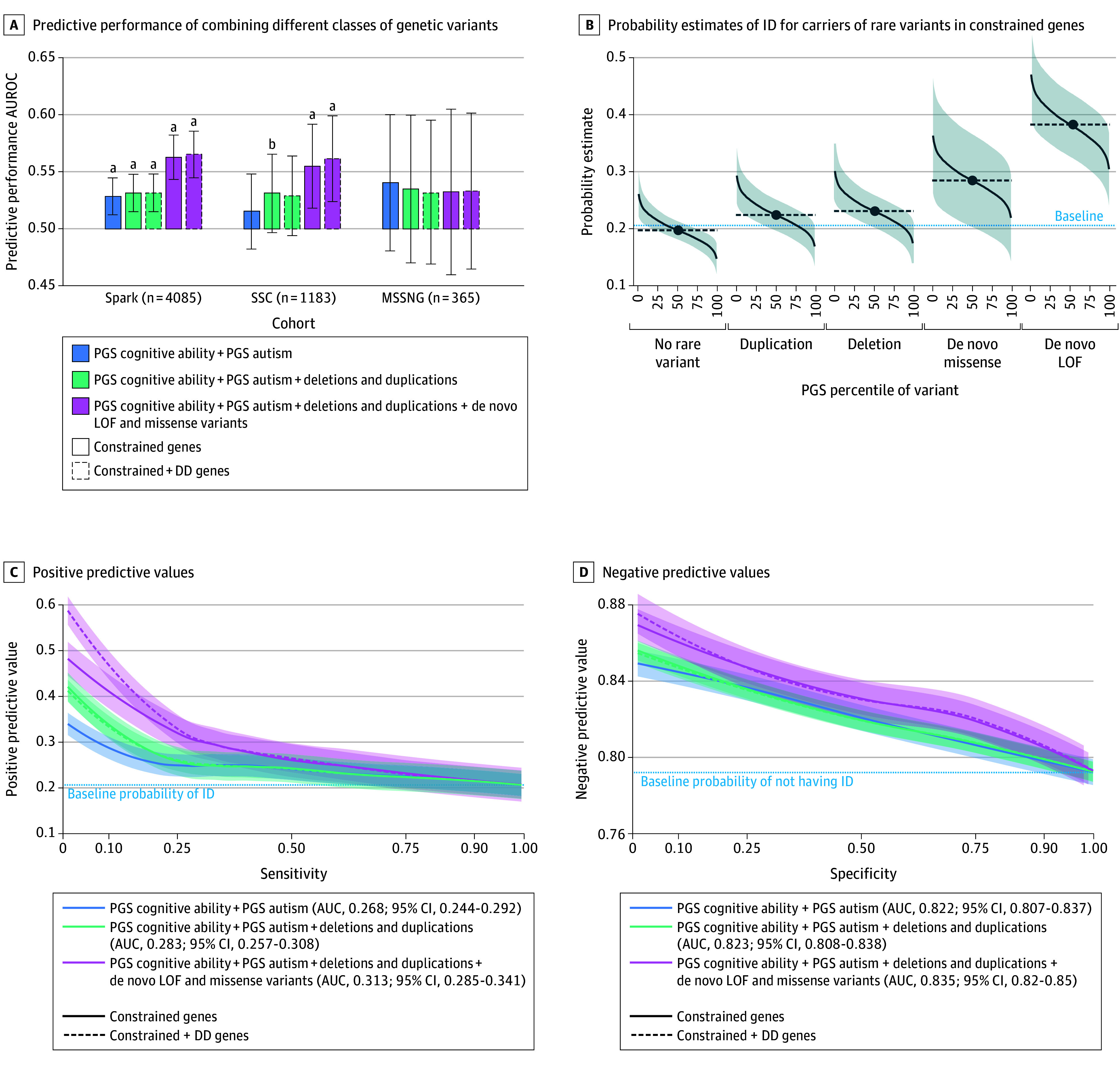
Genetic Analyses to Predict Intellectual Disability (ID) in Autistic Individuals A, Predictive performance of combining different classes of genetic variants, assessed using cross-validation in the Simons Foundation Powering Autism Research (SPARK) cohort and evaluated for generalizability in the Simons Simplex Collection (SSC) and MSSNG cohorts. The predictive performance of each model is tested using two complementary methods: the likelihood-ratio test, and the area under the curve (AUROC) with its 95% CI. Genetic variants were included sequentially into the models: polygenic scores (PGS) for cognitive ability and autism, deletions and duplications, and de novo loss-of-function (LOF) and missense variants. Rare variants were annotated based on constrained genes alone or combined with developmental disorder (DD) genes. Benjamini-Yekutieli adjustment for multiple comparisons was used. B, Probability estimates of ID for carriers of rare variants in constrained genes are shown (colored curve) as a function of their common variant background, measured by PGS for cognitive ability. Shaded areas representing 95% CIs. Dashed lines indicate the average probability of ID for carriers of each class of rare variant, as well as for individuals who do not carry any rare variant. The baseline probability (0.208) corresponds to the prevalence of ID in the total sample, irrespective of genetic variants. C, Positive predictive values achieved at different probability thresholds reflect the trade-off with the model’s sensitivity. For a sensitivity value of 10%, models integrating different classes of genomic variants yield progressively higher positive predictive values, 0.288 with PGS, 0.339 with adding deletions and duplications (0.334 with DD genes), and 0.411 with adding missense and LOF variants (0.468 with DD genes). Shaded areas represent 95% CIs. The area under the curve (AUC) reflects the overall ability to predict ID and should be compared with the sample’s baseline ID prevalence (20.8%). D, As a counterpart to panel C, negative predictive values achieved at different probability thresholds reflect a trade-off with the model’s specificity. The AUCs reflect the overall ability to predict the absence of ID, and should be compared with the sample’s baseline prevalence of not having ID (79.2%). ^a^*P* < .001. ^b^*P* < .01.

We first investigated if genetic variants alone could predict ID among autistic individuals (Figure 2A). In the training cohort (SPARK), all genetic models were statistically significant (likelihood ratio test, *P* = 1.1 × 10^−17^ for the model including all classes of variants) and we observed an increase in cross-validated predictive performance measured with the AUROC with sequentially incorporating the different classes of common and rare genetic variants into the model, all of which were significant, with *P* = .02 for deletions and duplications (*P* = .02 when including DD genes) and *P* = 1.4 × 10^−10^ for LOF and missense variants (*P* = 8.9 × 10^−15^ when including DD genes).

The predictive performance of the model combining cognitive ability PGS and autism PGS (both negatively correlated with ID) resulted in an AUROC of 0.529 (95% CI, 0.512-0.545). With the inclusion of constrained and DD gene deletions and duplications, this was 0.532 (95% CI, 0.515-0.548), and when also incorporating de novo LOF and missense variants, this was 0.565 (95% CI, 0.545-0.586).

#### Generalization to Different Cohorts

We tested whether the model’s predictive performance was generalizable to other cohorts with different ascertainment (Figure 2A and eTable 3 in Supplement 2). The model trained on SPARK was validated out-of-sample in SSC and MSSNG. The model’s performance was robust to resampling in SSC (AUROC, 0.562; 95% CI, 0.524-0.599; likelihood-ratio test *P* = 1.2 × 10^−6^) but not in the smaller MSSNG dataset (AUC, 0.533; 95% CI, 0.465-0.602; likelihood-ratio test *P* = .41).

#### Examining Model Predictions

We examined the probability estimates (Figure 2B) obtained from combining PGS for cognitive ability with rare genetic variants of different classes. Most carriers had only 1 gene impacted by LOF (97.9% [285 of 291] of carriers) or missense (99.2% [122 of 123]) variants. In contrast, CNVs are often multigenic: 36.3% of carriers (49 of 135) of deletions and 41.5% of carriers (81 of 195) of duplications had variants involving more than 1 constrained gene (SPARK data). Considering the additive effects of these variants,^23,53,54^ we estimated that a deletion or duplication impacting 3 or 4 constrained genes respectively, combined with a top-decile PGS, confers a similar probability of intellectual disability (approximately 34%) as a LOF variant with a bottom-decile PGS. In individuals with a higher probability due to a de novo LOF variant, the top and bottom decile PGS were associated with predicted probabilities of ID of less than 34.0% and greater than 43.0%, respectively, as compared with less than 17.1% and greater than 23.2% in those without any rare variant. In other words, combining rare and common variants improved discrimination between low vs high probabilities of ID.

#### Evaluating PPVs and NPVs

To evaluate the clinical relevance of this model, we examined trade-offs between PPVs and sensitivity, as well as between NPVs and specificity (eAppendix in Supplement 1), across the full range of combinations of genetic variants (Figure 2C and D).

We observed that the addition of each class of variant into the model (Figure 2C) consistently improved PPVs. Certain combinations of variants reached a PPV of 46.8%. In other words, 46.8% of the individuals predicted to have ID by the model had a diagnosis of ID (2-fold increase compared with the baseline prevalence of 20.8%). Such high-liability combinations of variants occurred in a small proportion of individuals, thus correctly identifying 10% (sensitivity) of individuals who will develop ID. Conversely, this curve illustrates that the integration of all genetic variants also increases the number of identified children among all those having ID (ie, sensitivity) from 6% when using only polygenic scores to 30% when incorporating all variants, while maintaining a stable PPV of approximately 30%.

Gains in NPVs (Figure 2D) were more modest. The NPVs reached 86.0%, as observed at the lowest 10% of specificity, compared with the 79.2% baseline prevalence of not having ID. This suggests that genetic variants, when used in isolation, are more effective at predicting the presence of ID (Figure 2C) than excluding it (Figure 2D).

Many variants (with mild to moderate effect sizes) integrated in our model would either not be reported by most diagnostic laboratories or be reported as variants of unknown significance. Therefore, we compared our model’s PPVs with that of variants that would currently be reported to clinicians as pathogenic by diagnostic laboratories. The carrier status of deletions or de novo LOF variants disrupting DD-associated genes^45^ had a PPV of 49.8%, similar to that of the model integrating all classes of variants, albeit with a 3-fold smaller sensitivity (3.3%) (eTable 4 in Supplement 2). This indicates that models integrating different classes of genetic variants achieve PPVs similar to those of currently reported pathogenic variants in a 3-fold larger group of individuals (ie, improved sensitivity).

#### Sensitivity Analyses

The eMethods in Supplement 1 showed that the model’s predictive performance was not influenced by environmental factors (ie, removing individuals with prenatal exposure to alcohol or drugs, oxygen supplementation at birth, intraventricular hemorrhage, meningitis, or encephalitis) (eTable 5 in Supplement 2), verbal abilities (ie, removing nonspeaking individuals) (eTable 5 in Supplement 2), changing predictive algorithm (random forest vs logistic regression) (eTable 6 in Supplement 2), or telescoping effects^55^ on milestones reporting (eTable 7 in Supplement 2).

### Integrating Genetic Variants and Developmental Milestones to Predict Outcome

When a child is referred for atypical development, clinicians commonly take into account developmental history to assess the probability of developing ID, based on normative data and their clinical experience. We aimed to evaluate the predictive informativeness of genetic test results on the background of this clinical information.

**Figure 3.**
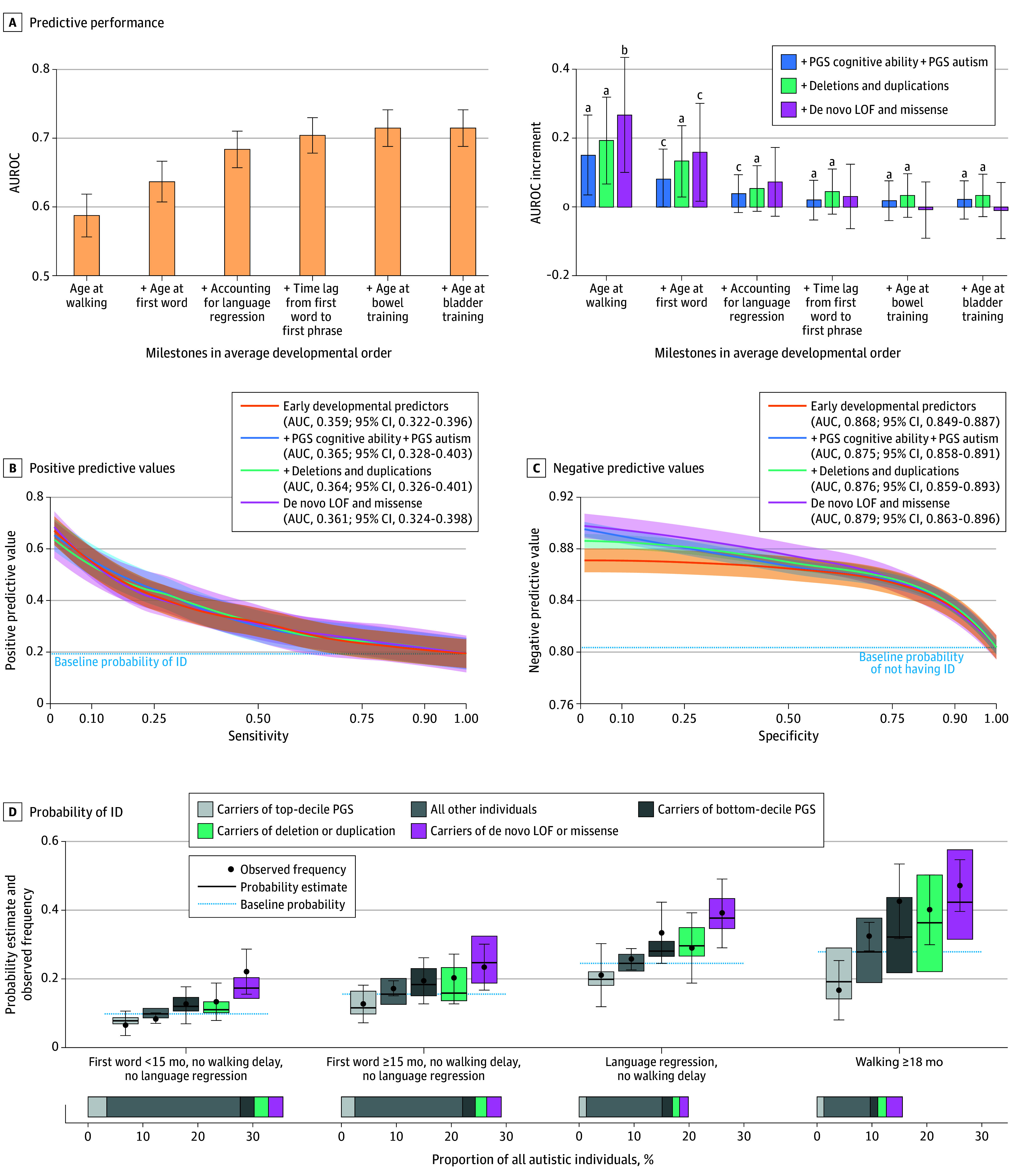
Combining Genetic Variants and Developmental Milestones to Predict Intellectual Disability (ID) in Autistic Individuals A, The left panel shows the area under the receiver operating characteristic (AUROC) curve of models sequentially adding milestones in the typical order of child development. The right panel shows to what extent genetic variants provide additional information when combined with milestones. Genetic variants were included sequentially into the models: polygenic scores (PGS) for cognitive ability and autism, deletions and duplications, de novo loss-of-function (LOF) and missense variants. *P* values were obtained from the likelihood ratio test and Benjamini-Yekutieli adjustment for multiple comparisons was used. B and C, Positive predictive values (PPVs) and negative predictive values (NPVs) achieved at different sensitivity and specificity thresholds, respectively. For a sensitivity value of 50%, 25%, and 10%, PPVs of developmental predictors are 0.317, 0.419, and 0.55, respectively. NPVs are higher for the model including PGS: at a specificity threshold of 10%, the model integrating PGS yields an NPV of 0.894, compared with 0.871 for early developmental predictors alone. D, Probability of ID in groups defined based on common reasons for clinical referral. The box plot represents the distribution of out-of-sample model predictions (median and IQR) The black dots and 95% CIs represent the observed frequencies of ID within the same group. The bottom panels indicate the percentage of autistic individuals assigned to each group. ^a^*P* < .001. ^b^*P* < .05. ^c^*P* < .01.

First, we tested the predictive value of developmental history, by sequentially including in a model the age of attaining 5 milestones, as well as the presence of language regression, in the order these typically occur (Figure 3A, right panel).^52^ As expected, the predictive performance increased as we added developmental milestones that are achieved at older ages, closer to the diagnosis of ID.

Then, we assessed the added predictive value of genetics (Figure 3A, left panel) at each developmental stage, compared with milestones alone. The added predictive value of genetic variants was contingent on the performance of milestones, such that it decreased when milestones occurring at an older age were included in the model, suggesting that genetic variants contribute more prediction early in development when fewer milestones have been achieved and less cumulative developmental information is available. We then focused on a model integrating the ages of walking and first words as these milestones are typically observed at the time of referral for autism assessment. The predictive performance was AUROC = 0.637 (95% CI, 0.608-0.667). The sequential additions of polygenic scores, copy number variants, and de novo coding variants into the model were respectively associated with increments in AUROC of 0.008 (likelihood-ratio test, *P* = 1.0 × 10^−3^), 0.013 (*P* = 1.6 × 10^−5^), and 0.016 (*P* = 9.3 × 10^−3^). This complete model had an AUROC of 0.653 (95% CI, 0.625-0.681).

#### Evaluating PPVs and NPVs

To understand the strengths and limitations of this model for clinical predictions, we examined PPVs and NPVs across combinations of genetic and developmental trajectories (Figures 3B and C). We expected that incorporating genetic information alongside developmental milestones would primarily improve PPVs, as clinicians generally consider the carrier status of a genetic variant more clinically significant than its absence. However, we observed that the addition of PGS to developmental milestones improved NPVs (Figure 3C). Certain combinations of variants (or their absence) and milestones reached a NPV of 89%.

#### Stratification of Probability Estimates

We aimed to assess predictive accuracy in clinical situations where children are referred for specific concerns. We, therefore, computed probability estimates within 4 groups that represent common reasons for referral to developmental clinics, such as delayed (>90th percentile) motor or language milestones or language regression.^52^

The observed frequencies of ID for each group were concordant with the probabilities of ID predicted by the model (Figure 2D). The stratification between low vs high probabilities provided by genetic variants was increased 2-fold in individuals with significant developmental delays (probabilities ranging from 16.7%-47.1%) compared with those without such delays (7.1%-22.1%).

#### Out-of-Sample Generalization

The predictive performances provided by the combination of developmental milestones and genetic variants were generalized to SSC (AUROC = 0.739; 95% CI, 0.694-0.784) and MSSNG (0.726; 95% CI, 0.639-0.813) (eTable 6 in Supplement 2).

### Differential Prediction of Cognitive and Adaptive Dimensions

The diverse adaptive and cognitive profiles of autistic individuals are more complex than the category of ID. Indeed, consistent with previous reports,^56,57^ we observed a nonlinear relationship between IQ and Vineland adaptive scales (eMethods in Supplement 1), which highlighted that a proportion of autistic individuals with typical-range IQ (>70) had greater impairment in adaptive functioning than would be expected based on their IQ.

**Figure 4.**
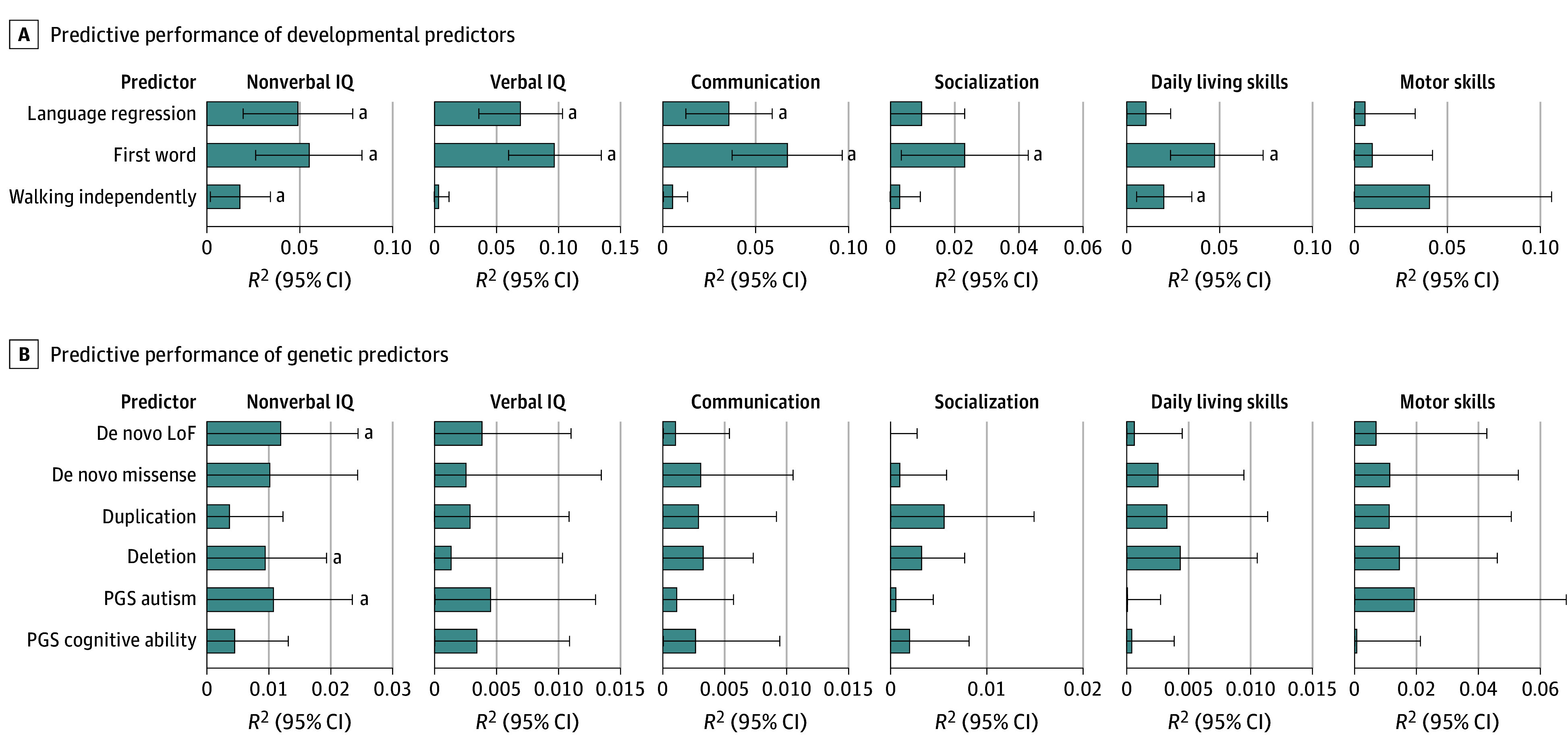
Differential Prediction of Adaptive and Cognitive Dimensions Using Genetic Variants and Developmental Milestones The predictive performance of developmental (A) and genetic (B) predictors varies across cognitive and adaptive dimensions, as shown by their distinct patterns of explained variance (*R*^2^). Significance is evaluated against the null model. All subscales and predictors were evaluated in the same sample of 1054 participants from the Simons Simplex Collection cohort, except for the optional subscale or motor skills, which were assessed in a subset of 160 participants where motor impairments were suspected by examiners. ^a^*P* < .05 (Benjamini-Yekutieli adjustment for multiple comparisons).

Therefore, we asked whether genetic variants and early milestones were more predictive of some defining features of ID rather than others: verbal and nonverbal IQ, as well as the 4 Vineland subscales of adaptive functioning (Figure 4 and eTable 8 in Supplement 2). We evaluated this in a subset of autistic individuals from the SSC sample. Significant milestones explained on average 4-fold more variance than genetics, with on average 4.8% of variance explained by milestones vs 1.1% for the genomic variants assessed in this study (including significant effects only). All classes of rare and common variants, as well as age of walking, were more predictive of nonverbal IQ, which is often difficult to assess in autistic individuals.^38^

## Discussion

Leveraging the broad spectrum of combinations of genetic variants and developmental milestones observed in autistic individuals, we developed models that provide PPVs of up to 55%, accurately identifying 10% of individuals who will develop ID. Although the addition of genetic variants to developmental milestones especially improved the identification of individuals who will not develop ID (NPVs), the ability to stratify the probabilities of ID using genetic variants was 2-fold greater in individuals with delayed milestones compared with those with typical development.

Our approach builds on previous studies showing that the effects of rare variants are modulated by an individual’s genetic background of common variants.^23,53,58,59^ This was previously shown in the 22q11.2 deletion, where the predictive power of PGS for ID was higher in carriers (due to an elevated baseline prevalence) compared with the general population.^53^ Here, we extended this approach to all autistic individuals. The inclusion of multiple combinations of rare and common variants—without any phenotypic information—resulted in a relative 2-fold increased probability, PPVs of 46.8% compared with a baseline probability of 20.8%.

Probability prediction in other medical fields such as cardiovascular health relies on quantitative tools that integrate multiple variables, each contributing modestly but collectively achieving clinically significant predictions.^60^ Next steps to improve predictive performances of neurodevelopmental outcomes will similarly require the integration of iterative assessments, comprehensive developmental scales,^13^ familial and environmental factors, as well as a saturated map of all genetic variants—each contributing incrementally to predictive performance. Although the heritability of ID related to additive and transmitted variants^61^ is high, a considerable portion of genetic variants contributing to ID remains unidentified. Current polygenic scores explain approximately 5% of variance in general cognitive ability,^62^ which is low compared with other traits (with similar heritability) such as height where association studies have reached saturation and provide PGS explaining up to 40% of variance.^63^ Improved knowledge of rare, common, as well as lesser known intermediate-frequency genetic variants involved in neurodevelopmental conditions, along with association studies performed across diverse ancestries, is expected to increase the predictive power of such models.

The pleiotropy of genetic variants, influencing multiple developmental phenotypes, such as autism, ID,^64^ as well as developmental milestones,^23^ may represent an opportunity to improve predictive models by using developmental milestones as proxies for currently unmeasured genomic liabilities (eg, unidentified gene-disrupting variants may be present in children with significantly delayed milestones). Conversely, we showed that genetic variants bring additional predictive information (albeit modest) compared with milestones alone. Although genetic variants alone led to greater gains in PPVs (Figure 2C), incorporating genetic data, particularly PGS, with developmental milestones improved NPVs (Figure 3C). This suggests that PPVs from genetic variants overlap with those from developmental milestones, whereas their NPV—specifically from polygenic scores—is more orthogonal to developmental milestones.

### Limitations

This study has some limitations. First, retrospective evaluation of milestones may be susceptible to recall biases or telescoping effects, where developmental milestones can be remembered by caregivers as more recent than they occurred.^55^ We found, however, no evidence of such effects. Such parental report mirrors current practices, where developmental history is gathered retrospectively by clinicians. Moreover, retrospective motor milestones have previously been shown to be reliable as compared to prospective inquiry.^65^ Second, models were trained and cross-validated on parent reports of a clinical diagnosis of ID in the SPARK cohort, and their out-of-sample validity were examined on IQ scores less than 70 in the SSC and MSSNG cohorts. Although these approaches differ, the consistency of results across datasets further highlights the generalizability of the models. Third, participation bias^66^ may have led to an overrepresentation of individuals without ID, including possibly those individuals who are carriers of variants in known autism/ID genes, because significant ID may exclude some individuals from an autism diagnosis and/or participation in research.^67^ This could reduce the predictive performance of models for predicting ID. Indeed, it was previously shown that in the SPARK cohort, more than 25% of autistic individuals carrying de novo LOF variants in a set of autism-associated genes had an IQ in the typical range.^24^ Fourth, due to the current underrepresentation of diverse ancestries in genomics research, it is likely that the models would underperform in diverse ancestries.^68^

## Conclusions

This prognostic study highlights the feasibility of using predictive models to assist clinicians in their assessment of children referred for autism. Models can provide families with probability estimates for different developmental trajectories and the corresponding levels of uncertainty tied to the interpretation of developmental milestones and genetic findings in the context of autism.

## Notes

Supplement 1.
eMethods.

eAppendix.

eReferences

Supplement 2. **eTable 1.** Comparison of the Clinical Diagnosis of Intellectual Disability With Cognitive and Adaptive Scales From Medical Records in SPARK **eTable 2.** Selection of Polygenic Scores **eTable 3.** Model Performance for Predicting Intellectual Disability Using Genetic Variants **eTable 4.** Comparison With the Predictive Performance or Carrier Status of Developmental Disorder Genes **eTable 5.** Other Risk Factors for Intellectual Disability and Confounding Factors in the Evaluation of ID **eTable 6.** Model Performance for Predicting Intellectual Disability Using Developmental Milestones and Genetic Variants **eTable 7.** Examination of Potential Confounding Effects of the Age at Assessment **eTable 8.** Performance for Predicting Intellectual Quotient and Vineland Adaptive Behavior Scales Using All Predictors

Supplement 3.
Data Sharing Statement.
