# Supplementary material for: Genomic and Developmental Models to Predict Cognitive and Adaptive Outcomes in Autistic Children
Source: JAMA Pediatr. 2025 Apr 21;179(6):655–65. doi: 10.1001/jamapediatrics.2025.0205 (PMC12012735; doi:10.1001/jamapediatrics.2025.0205)
Supplement: Supplement 1. — eMethods. eAppendix. eReferences [file jamapediatr-e250205-s001.pdf]

Supplementary Online Content

Bourque VR, Schmilovich Z, Huguet G, et al. Genomic and developmental models to predict cognitive and adaptive outcomes in autistic children. *JAMA Pediatr*. Published online April 21, 2025. doi:10.1001/jamapediatrics.2025.0205

eMethods.

eAppendix.

eReferences

This supplementary material has been provided by the authors to give readers additional information about their work.

© 2025 Bourque VR et al. *JAMA Pediatrics*.

eMethods.

Definition of Intellectual Disability

In the SPARK cohort, Intellectual Disability (ID) was defined based on a parent or caregiver report of the following: “Has child/dependent ever been diagnosed with intellectual disability or cognitive impairment (previously may have been referred to as mental retardation)”.

Intellectual Disability is a clinical diagnosis defined in the Diagnostic and Statistical Manual of Mental Disorders (DSM-5-TR) and the International Classification of Diseases (ICD-11), that integrates an evaluation of an individual’s functional abilities in activities of daily life (e.g. communication, social participation, independent living), as well as an evaluation of cognitive abilities (e.g. Intellectual Quotient, IQ, score). As such, it is not solely based on an IQ cut-off, but integrates other clinical data, as well as clinical judgement:

ICD-11: “In Disorders of Intellectual Development, a measure of intelligence quotient (IQ) is not an isolated diagnostic requirement to distinguish disorder from normality, but should be considered a proxy measure of the ‘significant limitations in intellectual functioning’ that partially characterize Disorders of Intellectual Development. IQ scores may vary as a result of the technical properties of the specific test being used, the testing conditions, and a variety of other variables and also can vary substantially over the individual’s development and life course. The diagnosis of Disorders of Intellectual Development should not be made solely based on IQ scores but must also include a comprehensive evaluation of adaptive behaviour.”

© 2025 Bourque VR et al. *JAMA Pediatrics*.

DSM-5-TR: “IQ test scores are approximations of conceptual functioning but may be insufficient to assess reasoning in real-life situations and mastery of practical tasks. For example, a person with deficits in intellectual functioning whose IQ score is somewhat above 65–75 may nevertheless have such substantial adaptive behavior problems in social judgment or other areas of adaptive functioning that the person’s actual functioning is clinically comparable to that of individuals with a lower IQ score. Thus, clinical judgment is important in interpreting the results of IQ tests, and using them as the sole criteria for the diagnosis of an intellectual developmental disorder is insufficient.”

We included (eTable 1) a comparison of the clinical diagnosis of Intellectual Disability with rating scales in SPARK, in a subset of participants for whom cognitive (Intellectual Quotient, up to n=326) and/or adaptive (Vineland Adaptive Behavior Scales, up to n=1,275) scales were extracted from the medical records by researchers.[1]

In the SSC and MSSNG cohorts, due to unavailability of the clinical diagnosis for most of the participants, Intellectual Disability was defined by an IQ < 70 (i.e. more than 2 standard deviations below age norms), to reflect common research practices.[2]

Developmental milestones

Milestones were recorded as the age of attainment in months, except for language regression, which was binary-coded. For SPARK, milestones were collected through the Background History Questionnaire filled by a caregiver or independent adult. For SSC and MSSNG, milestones were collected through the Autism Diagnostic Interview-Revised.[3] The selected milestones are attained on average in the general population before 4 years of age[4], and language regression occurs in a proportion of

© 2025 Bourque VR et al. *JAMA Pediatrics*.

autistic individuals on average by 2 years of age.[5] Thus, participants had aged past this period at time of assessment (≥6 years old), which consequently allowed for complete records.

The ages at attaining the milestones were included as predictors within a multiple logistic regression. Previous research suggests that the relationship between the age at first word and IQ may be different in individuals with language regression[6], therefore we included an interaction term between those two predictors. To include a maximum of information from the age at first phrase whilst also accounting for its collinearity (r=0.72) with the age at first word, we calculated the time lag between first word and first phrase, which is supported by previous accounts of a language acquisition plateau in a proportion of autistic individuals.[6]

Genetic quality control and imputation

Genomic analyses were obtained either by single nucleotide polymorphism (SNP) genotyping (Illumina 1Mv1, 1Mv3, or Omni2.5 arrays for SSC; Illumina Infinium Global Screening Array-24 for SPARK) or by Whole-Genome Sequencing (WGS; we included samples sequenced with Illumina HiSeq, HiSeq 2500, or HiSeq X for MSSNG). In the case of WGS, we used PLINK v1.9[7] to convert the pVCF files into genotyping data format[8]. Using bcftools v1.13[9] we removed indels, selected biallelic sites, and normalised the SNP dataset.

We performed quality control for each genetic sequencing technology separately using established criteria[10] with PLINK v1.9.[7] In brief, we removed individuals with genotyping rate <95%, excessive heterozygosity (±3 standard deviations from the mean), sample missingness >0.02, mismatch in reported and genetic sex, or families with Mendelian errors >5%. We removed SNPs with a call rate <98%, a minor allele

© 2025 Bourque VR et al. *JAMA Pediatrics*.

frequency (MAF) <1%, deviation from Hardy-Weinberg Equilibrium ($P < 1\times10^{-6}$), or >10% Mendelian error rate.

We assessed the population genetic structure using KING[11]. Considering that the current genome-wide association study (GWAS) summary statistics are primarily Euro-centric, we included individuals of comparable genetic background, i.e. with ≥85% probability of genetically inferred European ancestry.

We imputed the missing SNPs using the 1000 Genomes Project phase 3 (1KGP3) reference panel[11] on the Sanger Imputation Server.[12] We retained loci that were measured across all technologies. We merged the imputed files, converted them to PLINK files, and we removed SNPs with a poor imputation quality metric (INFO≥0.3), multiallelic (>2) variants, MAF <5%, call rate <98%, or significant deviation from Hardy-Weinberg Equilibrium ($P < 5\times10^{-7}$).

To account for population stratification within the final sample, we computed the top 10 ancestry principal components (PCs) using the mds function in the KING population structure inference tool, with the 1000 Genomes Project data as a reference population.[11]

Polygenic scores

To compute the polygenic scores (PGS), we used GWAS summary statistics for autism and 11 traits genetically correlated to autism. These traits were previously selected from a total of 234 traits tested for genetic correlations with autism, as presented in the autism GWAS publication.[13] We reasoned that given their correlation with autism, these genetic liabilities could potentially also be relevant with regards to the heterogeneity of outcomes among autistic individuals. For the autism

© 2025 Bourque VR et al. *JAMA Pediatrics*.

GWAS statistics, we included only the iPsych sample, ensuring no overlap between the base GWAS samples and the target samples of this study.

We computed PGS using polygenic likelihood scoring with continuous shrinkage (PRS-CS), which is one of the polygenic scoring strategies that yields the highest variance explained.[14] To remove potential confounding effects of population structure, we residualised the top 10 ancestry principal components from each PGS within each cohort.

To select the most predictive combination among the 12 PGS, we used the technique of forward feature selection in Scikit-learn[15] with 10-fold cross validation in the SPARK dataset, because these were partly collinear and varied significantly in their predictive performance for ID. The selection criterion was to optimise predictive performance assessed by the AUC ROC, with a 1% improvement threshold for selection. This led us to retain for the analyses the two most predictive PGS: that for cognitive ability[16] followed by that for autism[13] (eTable 2), which we combined in an additive linear model, as previously reported.[17] In addition, we confirmed that this selection of PGS was robust to a different method, using a backward feature selection algorithm (eTable 2).

Copy number variants

The methods used for copy number variants (CNV) calling and filtering have been described in previous publications.[18–20] We identified CNVs from SNP arrays in SPARK and SSC using two independent pipelines, PennCNV[21] and QuantiSNP[22], with the following parameters: number of consecutive probes ≥3, CNV size ≥1Kb, likelihood score ≥15. A consensus from these two pipelines was obtained using CNVision[23], such as to minimise false discoveries. We concatenated adjacent CNVs

© 2025 Bourque VR et al. *JAMA Pediatrics*.

of the same type using the following criteria: gap between CNVs ≤150 kb, size of the CNVs ≥1Kb, and number of probes ≥3. We removed additional artefacts using a previously published pipeline,[18–20] we retained CNVs with call rate ≥95%, log R ratio standard deviation <0.35, B-allele frequency standard deviation <0.08, |wave factor|<0.05, and a likelihood score ≥30 for at least one detection algorithm. We remove arrays with a suspiciously high number of detections, ≥50 for low-resolution arrays (<1 million probes) and ≥ 200 for high-resolution arrays (≥1 million probes). We retained CNVs with <50% overlap with segmental duplications or centromeric regions.

We used CNVs called from Illumina whole genome sequencing (WGS) provided by MSSNG, as previously published.[24,25] Illumina sequencing was conducted using the HiSeq, HiSeq 2500, or HiSeq X platforms. CNVs ≥1 kb were identified using ERDS[26] and CNVnator[27] pipelines. CNVs were retained if detected by both pipelines with ≥50% reciprocal overlap, and had <70% overlap with assembly gaps, segmental duplications, or centromeric regions. Adjacent CNVs of the same type were concatenated if they were separated by a gap in the genome assembly or by a small region, provided the combined length of individual CNVs represented >70% of the merged CNV length.

For consistency across all technologies and cohorts, we retained CNVs ≥50 kb that overlapped ≥10 probes in each array technology. We retained autosomal CNVs with size <10Mb. CNVs were annotated with Gencode V19 (hg19) using ENSEMB,[28] assigning coding genes of which all isoforms were fully encompassed.

© 2025 Bourque VR et al. *JAMA Pediatrics*.

De novo coding variants

We used previously published calls of *de novo* coding variants for SPARK, SSC[29] and MSSNG[30]. We included all loss-of-function (LoF) variants, as well as missense variants with high variant-level Missense PolyPhen Constraint (MPC≥2).[31,32]

Cognitive and adaptive scales

Verbal and non-verbal IQ scores were assessed using WISC-IV, DAS-II E-Y, DAS-II S-A, or WASI-I, and Vineland Adaptive Behavior Scales[33] were assessed in n=1,054 participants from the SSC sample. Among these, the motor skills subscale was assessed for a subset of n=160 participants for whom the examiners suspected motor impairments. To assess the correlations among these subscales, quantile regressions using the method of fractional polynomials[34] were fitted with the GAMLSS package[35]. We evaluated the predictive performance for each genetic and developmental predictor as the variance explained (R^2), while subtracting that from covariates (sex and first 10 genetic principal components).

Models

To determine the cumulative and incremental predictive contributions of variables, we used multiple logistic regression, sequentially adding variables in a predetermined order, as follows.

(A) Genetic models: We integrated polygenic scores (cognitive ability and autism), followed by CNVs (deletions and duplications) and *de novo* coding variants (LoF and missense variants). Sex was included as a covariate in all models, and we then adjusted the performance estimates (AUC ROC) to specifically reflect the contributions attributable solely to genetic variables.

© 2025 Bourque VR et al. *JAMA Pediatrics*.

(B) Developmental models: Developmental milestones were incorporated sequentially based on the average age at which they are typically attained in child development[36], and including sex as a covariate.

(C) Integrated Models: Following the approach used in other medical fields for "integrated risk models",[37,38] we combined genetic variants in the same models with developmental milestones. We evaluated the increase in predictive performance resulting from the sequential addition of each genetic variable.

As a secondary analysis, we compared the results with that of a random forest algorithm[15], to investigate whether this could handle better than logistic regression the multiple predictor variables as well as their potential interactions.

Metrics of predictive performance

To ensure that models can be utilised across various settings, we chose not to set a specific probability threshold for predicting a "case". This approach avoids oversimplifying nuanced probability predictions into mere classification and allows the probability decision threshold to be adjusted according to other factors in the process of shared decision making. Therefore, we chose predictive ability metrics that do not rely on a predetermined decision threshold: We assessed (1) the area under the ROC curve (AUC ROC)[39], which evaluates how well the model distinguishes cases from non-cases, (2) the area under the PPV-sensitivity curve[40] (also known as the precision-recall curve), and (3) the area under the NPV-specificity curve. To accurately estimate out-of-sample predictive performance, we calculated these metrics independently within each of the 10 folds in the SPARK cohort's cross-validation, thus ensuring that the training and validation data remained distinct, and we then computed the average performance across all folds. To obtain

© 2025 Bourque VR et al. *JAMA Pediatrics*.

PPV-sensitivity and NPV-specificity curves, we computed those curves for each fold in the SPARK dataset, calculated the mean across all folds within 100 sensitivity or specificity bins, and then adjusted locally estimated scatterplot smoothing (LOESS) regressions to the results using the GAMLSS package.[35] Finally, we tested out-of-sample prediction of the model trained on the complete SPARK sample to generalise on SSC and MSSNG samples. The functions used for model training and evaluation were made available in a public repository: https://github.com/vrbourque/prediction-metrics/.

Sensitivity analyses

We assessed whether other factors may have impacted the models' performance in predicting ID. We evaluated the impact of removing individuals who had prenatal exposure to alcohol or drugs, oxygen supplementation at birth, intraventricular haemorrhage, meningitis, or encephalitis (6.1% of individuals in SPARK). Additionally, because cognitive assessments of non-speaking individuals may not accurately reflect their cognitive abilities, we assessed the impact of removing non-speaking individuals (10.8% of individuals in SPARK).

To determine if including interactions and potential nonlinear relationships would change predictions, we replaced the logistic regression with a Random Forest algorithm and compared the predictive performance.

We examined whether the age at assessment may have impacted the reporting of the age at attaining milestones ("telescoping effects")[41] by testing all associations between milestones included in the analyses and the age at assessment, using a linear model adjusted for sex. We also tested quadratic effects for age.

© 2025 Bourque VR et al. *JAMA Pediatrics*.

Correlation matrix of standardised cognitive and adaptive scores

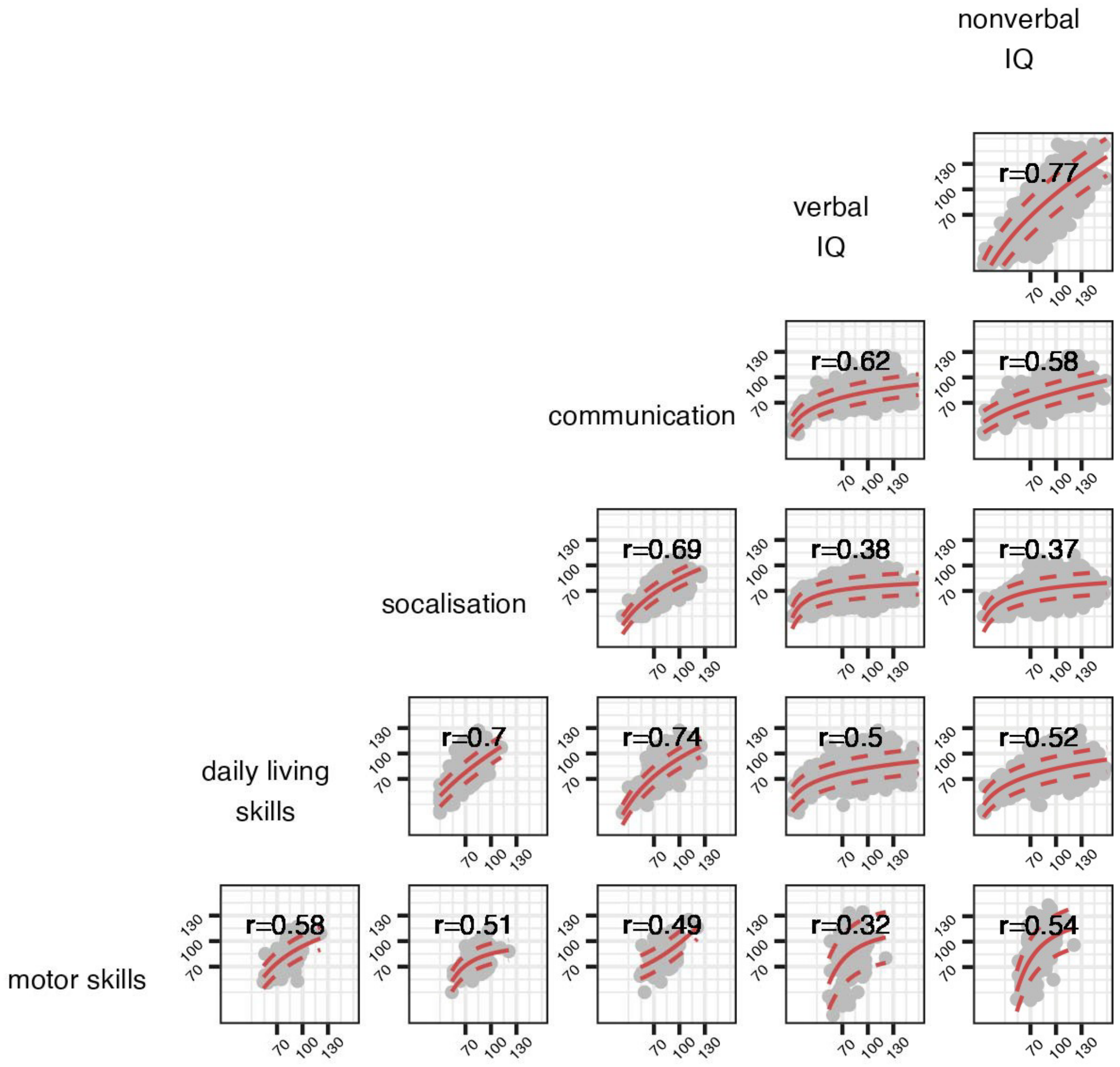

This figure displays the correlations between standardised scores of cognitive and adaptive dimensions, measured by IQ and Vineland adaptive behaviour scales, respectively. Red lines represent the median, 10th, 50th, and 90th percentiles of the non-linear trend computed using GAMLSS. The Spearman correlation matrix indicates non-linear relationships ranging from r=0.32 to 0.77. X and Y-axis values are standardised scores for each subscale with mean 100, and standard deviation 15. The scores were evaluated in a sample of 1,054 participants from the SSC

© 2025 Bourque VR et al. *JAMA Pediatrics*.

cohort, except for the optional subscale or motor skills, which was assessed in a subset of 160 participants where motor impairments were suspected by examiners.

© 2025 Bourque VR et al. *JAMA Pediatrics*.

eAppendix.

Box. Definition of key technical terms

Out-of-sample predictive performance: the ability of a model - developed using data from one sample of individuals - to accurately predict an outcome (in this study, intellectual disability) in newly ascertained individuals, based on their clinical characteristics (eg, developmental) and/or laboratory testing (eg, genetic).

Cross-validation: a technique for estimating a model's out-of-sample predictive performance within a single sample, by randomly dividing that sample into multiple groups of individuals and repeatedly training and evaluating the model's performance on these distinct groups.

Generalisability: a model's out-of-sample predictive performance in an entirely new sample, which might consist of individuals ascertained differently and in another clinical center.

Area under the Receiver Operating Characteristic curve (AUC ROC): a gold-standard measure of a model's global performance in providing probability estimates that accurately distinguish individuals that will develop an outcome from those that will not.

Positive Predictive Value (PPV): the proportion of individuals who develop an outcome, among all those predicted by the model to develop this outcome.

Negative Predictive Value (NPV): the proportion of individuals who do not develop an outcome, among all those predicted by the model to not develop this outcome.

Sensitivity: the proportion of individuals that were correctly identified by the model, among all those who develop an outcome. Also known as the true positive rate.

Specificity: the proportion of individuals that were correctly identified by the model, among all those who actually do not develop an outcome. Also known as the true negative rate.

Probability stratification: the ability to provide a range of probability estimates of an outcome according to each individual's clinical characteristics (eg, developmental) and/or laboratory testing (eg, genetic).

© 2025 Bourque VR et al. *JAMA Pediatrics*.

eReferences

1. Fombonne E, Coppola L, Mastel S, O'Roak BJ. Validation of autism diagnosis and clinical data in the SPARK cohort. *J Autism Dev Disord*. 2022;52(8):3383-3398.

2. Kuo SS, van der Merwe C, Fu JM, et al. Developmental Variability in Autism Across 17 000 Autistic Individuals and 4000 Siblings Without an Autism Diagnosis: Comparisons by Cohort, Intellectual Disability, Genetic Etiology, and Age at Diagnosis. *JAMA Pediatr*. 2022;176(9):915-923.

3. Lord C, Rutter M, Le Couteur A. Autism Diagnostic Interview-Revised: a revised version of a diagnostic interview for caregivers of individuals with possible pervasive developmental disorders. *J Autism Dev Disord*. 1994;24(5):659-685.

4. Sheldrick RC, Schlichting LE, Berger B, et al. Establishing New Norms for Developmental Milestones. *Pediatrics*. 2019;144(6). doi:10.1542/peds.2019-0374

5. Barger BD, Campbell JM, McDonough JD. Prevalence and onset of regression within autism spectrum disorders: a meta-analytic review. *J Autism Dev Disord*. 2013;43(4):817-828.

6. Gagnon D, Zeribi A, Douard É, et al. Bayonet-shaped language development in autism with regression: a retrospective study. *Mol Autism*. 2021;12(1):35.

7. Purcell S, Neale B, Todd-Brown K, et al. PLINK: a tool set for whole-genome association and population-based linkage analyses. *Am J Hum Genet*. 2007;81(3):559-575.

8. Hofmeister RJ, Ribeiro DM, Rubinacci S, Delaneau O. Accurate rare variant phasing of whole-genome and whole-exome sequencing data in the UK Biobank. *Nat Genet*. 2023;55(7):1243-1249.

9. Danecek P, Bonfield JK, Liddle J, et al. Twelve years of SAMtools and BCFtools. *Gigascience*. 2021;10(2). doi:10.1093/gigascience/giab008

10. Marees AT, de Kluiver H, Stringer S, et al. A tutorial on conducting genome-wide association studies: Quality control and statistical analysis. *Int J Methods Psychiatr Res*. 2018;27(2):e1608.

11. Manichaikul A, Mychaleckyj JC, Rich SS, Daly K, Sale M, Chen WM. Robust relationship inference in genome-wide association studies. *Bioinformatics*. 2010;26(22):2867-2873.

12. McCarthy S, Das S, Kretzschmar W, et al. A reference panel of 64,976 haplotypes for genotype imputation. *Nat Genet*. 2016;48(10):1279-1283.

13. Grove J, Ripke S, Als TD, et al. Identification of common genetic risk variants for autism spectrum disorder. *Nat Genet*. 2019;51(3):431-444.

© 2025 Bourque VR et al. *JAMA Pediatrics*.

14. Pain O, Glanville KP, Hagenaars SP, et al. Evaluation of polygenic prediction methodology within a reference-standardized framework. *PLoS Genet*. 2021;17(5):e1009021.

15. Pedregosa F, Varoquaux G, Gramfort A, et al. Scikit-learn: Machine Learning in Python. *J Mach Learn Res*. 2011;abs/1201.0490. doi:10.5555/1953048.2078195

16. Savage JE, Jansen PR, Stringer S, et al. Genome-wide association meta-analysis in 269,867 individuals identifies new genetic and functional links to intelligence. *Nat Genet*. 2018;50(7):912-919.

17. Antaki D, Guevara J, Maihofer AX, et al. A phenotypic spectrum of autism is attributable to the combined effects of rare variants, polygenic risk and sex. *Nat Genet*. 2022;54(9):1284-1292.

18. Huguet G, Renne T, Poulain C, et al. Effects of gene dosage on cognitive ability: A function-based association study across brain and non-brain processes. *Cell Genom*. 2024;4(12):100721.

19. Huguet G, Schramm C, Douard E, et al. Genome-wide analysis of gene dosage in 24,092 individuals estimates that 10,000 genes modulate cognitive ability. *Mol Psychiatry*. 2021;26(6):2663-2676.

20. Huguet G, Schramm C, Douard E, et al. Measuring and Estimating the Effect Sizes of Copy Number Variants on General Intelligence in Community-Based Samples. *JAMA Psychiatry*. 2018;75(5):447-457.

21. Wang K, Li M, Hadley D, et al. PennCNV: an integrated hidden Markov model designed for high-resolution copy number variation detection in whole-genome SNP genotyping data. *Genome Res*. 2007;17(11):1665-1674.

22. Colella S, Yau C, Taylor JM, et al. QuantiSNP: an Objective Bayes Hidden-Markov Model to detect and accurately map copy number variation using SNP genotyping data. *Nucleic Acids Res*. 2007;35(6):2013-2025.

23. Sanders SJ, Ercan-Sencicek AG, Hus V, et al. Multiple recurrent de novo CNVs, including duplications of the 7q11.23 Williams syndrome region, are strongly associated with autism. *Neuron*. 2011;70(5):863-885.

24. Trost B, Walker S, Wang Z, et al. A comprehensive workflow for read depth-based identification of copy-number variation from whole-genome sequence data. *Am J Hum Genet*. 2018;102(1):142-155.

25. Trost B, Thiruvahindrapuram B, Chan AJS, et al. Genomic architecture of autism from comprehensive whole-genome sequence annotation. *Cell*. 2022;185(23):4409-4427.e18.

26. Zhu M, Need AC, Han Y, et al. Using ERDS to infer copy-number variants in high-coverage genomes. *Am J Hum Genet*. 2012;91(3):408-421.

27. Abyzov A, Urban AE, Snyder M, Gerstein M. CNVnator: an approach to discover, genotype, and characterize typical and atypical CNVs from family and

© 2025 Bourque VR et al. *JAMA Pediatrics*.

population genome sequencing. *Genome Res*. 2011;21(6):974-984.

28. Martin FJ, Amode MR, Aneja A, et al. Ensembl 2023. *Nucleic Acids Res*. 2023;51(D1):D933-D941.

29. Zhou X, Feliciano P, Shu C, et al. Integrating de novo and inherited variants in 42,607 autism cases identifies mutations in new moderate-risk genes. *Nat Genet*. 2022;54(9):1305-1319.

30. C Yuen RK, Merico D, Bookman M, et al. Whole genome sequencing resource identifies 18 new candidate genes for autism spectrum disorder. *Nat Neurosci*. 2017;20(4):602-611.

31. Samocha KE, Kosmicki JA, Karczewski KJ, et al. Regional missense constraint improves variant deleteriousness prediction. *bioRxiv*. Published online June 12, 2017:148353. doi:10.1101/148353

32. Satterstrom FK, Kosmicki JA, Wang J, et al. Large-Scale Exome Sequencing Study Implicates Both Developmental and Functional Changes in the Neurobiology of Autism. *Cell*. 2020;180(3):568-584.e23.

33. Sparrow S, Cicchetti D, McColl E. Vineland Adaptive Behavior Scales Interview Edition expanded form manual. Published online 2015.

34. Royston P, Altman D. Regression using fractional polynomials of continuous covariates: parsimonious parametric modelling. *Insur Math Econ*. 1994;2:165-166.

35. Rigby RA, Stasinopoulos DM. Generalized Additive Models for Location, Scale and Shape. *J R Stat Soc Ser C Appl Stat*. 2005;54(3):507-554.

36. Dosman CF, Andrews D, Goulden KJ. Evidence-based milestone ages as a framework for developmental surveillance. *Paediatr Child Health*. 2012;17(10):561-568.

37. Elliott J, Bodinier B, Bond TA, et al. Predictive Accuracy of a Polygenic Risk Score–Enhanced Prediction Model vs a Clinical Risk Score for Coronary Artery Disease. *JAMA*. 2020;323(7):636-645.

38. Hujoel MLA, Loh PR, Neale BM, Price AL. Incorporating family history of disease improves polygenic risk scores in diverse populations. *Cell Genom*. 2022;2(7). doi:10.1016/j.xgen.2022.100152

39. Bradley AP. The use of the area under the ROC curve in the evaluation of machine learning algorithms. *Pattern Recognit*. 1997;30(7):1145-1159.

40. Inouye M, Abraham G, Nelson CP, et al. Genomic Risk Prediction of Coronary Artery Disease in 480,000 Adults: Implications for Primary Prevention. *J Am Coll Cardiol*. 2018;72(16):1883-1893.

41. Ozonoff S, Li D, Deprey L, Hanzel EP, Iosif AM. Reliability of parent recall of symptom onset and timing in autism spectrum disorder. *Autism*.

© 2025 Bourque VR et al. *JAMA Pediatrics*.

2018;22(7):891-896.

© 2025 Bourque VR et al. *JAMA Pediatrics*.
