# Supplementary material for: Genomic and Developmental Models to Predict Cognitive and Adaptive Outcomes in Autistic Children
Source: JAMA Pediatr. 2025 Apr 21;179(6):655–65. doi: 10.1001/jamapediatrics.2025.0205 (PMC12012735; doi:10.1001/jamapediatrics.2025.0205)
Supplement: Supplement 3. — Data Sharing Statement. [file jamapediatr-e250205-s003.pdf]

Data Sharing Statement

Bourque. Genomic and Developmental Models to Predict Cognitive and Adaptive Outcomes in Autistic Children. *JAMA Pediatr*. Published April 21, 2025.
doi:10.1001/jamapediatrics.2025.0205

Data
Data available: Yes
Data types: Deidentified participant data
How to access data: Approved researchers can obtain the SPARK and SSC population datasets described in this study by applying at https://base.sfari.org and to the MSSNG dataset at https://research.mss.ng/.
When available: With publication

Supporting Documents
Document types: None

Additional Information
Who can access the data: Approved researchers can obtain the SPARK and SSC population datasets described in this study by applying at https://base.sfari.org and to the MSSNG dataset at https://research.mss.ng/.
Types of analyses: Approved researchers can obtain the SPARK and SSC population datasets described in this study by applying at https://base.sfari.org and to the MSSNG dataset at https://research.mss.ng/.
Mechanisms of data availability: Approved researchers can obtain the SPARK and SSC population datasets described in this study by applying at https://base.sfari.org and to the MSSNG dataset at https://research.mss.ng/.
